# Supplementary material for: The coupling coordination relationship between sports industry agglomeration and economic resilience in the Yangtze River Delta region
Source: PLoS One. 2024 May 24;19(5):e0302356. doi: 10.1371/journal.pone.0302356 (PMC11125539; doi:10.1371/journal.pone.0302356)
Supplement: Attachment — Submitted filename: Response to Reviewers.doc [file pone.0302356.s006.doc]

Dear Reviewers':

Thank you for your letter and for the reviewers' comments concerning our manuscript entitled “The coupling coordination relationship between sports industry agglomeration and economic resilience in the Yangtze River Delta region." (Manuscript Number: PONE-D-23-37782). Those comments are all valuable and very helpful for revising and improving our paper, as well as providing important guidance for our further research. We have studied these comments carefully and have made corrections, which we hope will meet with approval. The words in blue are the changes I have made to the manuscript. The main corrections in the paper and the point-by-point responses to the reviewers' comments are as follows:

**Response to Reviewer #1**

1. **However, I noticed a minor writing issue. For instance, there are several instances of "between the two" in this manuscript, which seems informal and incomplete. I suggest modifying it to "between the two systems" or using other pronouns or abbreviations.**

**Response:** We thank the reviewer for this comment, and we strongly agree with the reviewers. At the same time, we have revised them to "between the two systems", words in blue are the change I have made in the file of “Revised Manuscript with Track Changes”.

**2. Furthermore, as someone equally interested in system coupling and the Yangtze River Basin, I recommend that the author consider referencing and citing** [**https://doi.org/10.3389/fevo.2023.1148868**](https://doi.org/10.3389/fevo.2023.1148868)

**Response:** Thanks for reviewer’s comments, and we strongly agreed with that the entropy method does not require expert intervention and can effectively eliminate human subjective influence, so we cited the article of “Regional sustainability: Pressures and responses of tourism economy and ecological environment in the Yangtze River basin, China” **in line 224-225.** In addition, The order of the references 44 to 52 was also refined.

Special thanks to you for your good comments. We tried our best to improve the manuscript as your suggestion. And here we did not list all the changes but marked all the amends in revised paper.

We appreciate for your warm work earnestly, and hope that the correction will meet with approval. Thank you for the kind advice again. If you have any questions, please contact us without hesitate.

Yours sincerely,

Hongyu MA

1, April, 2024

School of Sports and Health, Nanning Normal University

Guangxi, 530100, P. R. China

Tel:86-14795492349

Email: mhy@nnnu.edu.cn
